# Combining Spin-Seebeck and Nernst Effects in Aligned MnBi/Bi Composites

**DOI:** 10.3390/nano10102083

**Published:** 2020-10-21

**Authors:** Brandi L. Wooten, Koen Vandaele, Stephen R. Boona, Joseph P. Heremans

**Affiliations:** 1 Department of Materials Science and Engineering, The Ohio State University, Columbus, OH 43210, USA; wooten.120@osu.edu (B.L.W.); boona.1@osu.edu (S.R.B.); 2 Department of Mechanical and Aerospace Engineering, The Ohio State University, Columbus, OH 43210, USA; kovdaele@gmail.com; 3 Center for Electron Microscopy and Analysis, The Ohio State University, Columbus, OH 43212, USA; 4 Department of Physics, The Ohio State University, Columbus, OH 43210, USA

**Keywords:** spin-Seebeck effect, anomalous Nernst effect, thermoelectric composite, MnBi

## Abstract

The spin-Seebeck effect (SSE) is an advective transport process traditionally studied in bilayers composed of a ferromagnet (FM) and a non-magnetic metal (NM) with strong spin-orbit coupling. In a temperature gradient, the flux of magnons in the FM transfers spin-angular momentum to electrons in the NM, which by the inverse spin-Hall effect generates an SSE voltage. In contrast, the Nernst effect is a bulk transport phenomenon in homogeneous NMs or FMs. These effects share the same geometry, and we show here that they can be added to each other in a new combination of FM/NM composites where synthesis via in-field annealing results in the FM material (MnBi) forming aligned needles inside an NM matrix with strong spin-orbit coupling (SOC) (Bi). Through examination of the materials’ microstructural, magnetic, and transport properties, we searched for signs of enhanced transverse thermopower facilitated by an SSE contribution from MnBi adding to the Nernst effect in Bi. Our results indicate that these two signals are additive in samples with lower MnBi concentrations, suggesting a new way forward in the study of SSE composite materials.

## 1. Introduction

The spin-Seebeck effect (SSE) is the most recent addition to the family of spin-related thermal effects [1]. It involves the application of a temperature gradient to a metal [1], semiconductor [2], or insulating [3] ferromagnet (FM) or antiferromagnet [4]. It also has been observed in a diamagnetic semiconductor with electrons spin-polarized by an external field [5]. In insulating FMs, quantized fluctuations of localized electron magnetic moments known as magnons become thermally excited, resulting in a spin current proportional to the magnitude of the temperature gradient [6]. This spin current is directed towards an adjacent layer of a non-magnetic metal (NM) evaporated as a thin film onto the FM. The spin current spin-polarizes conduction electrons in the NM by conservation of spin-angular momentum [6]. Because the NM is chosen from a metal with strong spin-orbit coupling (SOC), the presence of spin-polarized electrons gives rise to an inverse spin-Hall effect (ISHE) electric field, thereby converting thermal energy into usable electric energy [6]: this is the SSE, an advective process whereby the heat first drives magnons, which in turn confer angular momentum to electrons. The ordinary (ONE) and anomalous (ANE) Nernst effects are non-advective transport processes whereby heat directly drives an electron flux, which is then deflected sideways either by a Lorentz force (the ONE in non-magnetic metals and semiconductors) or the presence of a Berry phase or skew scattering (the ANE in metallic FMs) [7]. Both ONE and ANE occur in homogeneous materials in the same geometry as the SSE, but they arise only in electrically conducting FMs. In the SSE, ANE, and ONE, the temperature gradient, magnetic field, and resulting electric field vectors are orthogonal to each other. Most recently, a possible advective component may have been identified in the ANE of certain FM metals via magnon drag in the case of Fe [8], or via magnon drag aided by a strong SOC contribution in MnBi [9]. This last contribution to the ANE can be thought of as a self-SSE in which a single homogeneous material provides both FM and NM qualities to achieve a transverse voltage or thermopower without the need for transfer across a heterostructure interface.

The traditional configuration for SSE devices is a planar thin film of NM on an FM substrate as described above. However, Boona et al. [10] have shown that it is possible to combine the ANE and SSE in composite materials where the NM/FM interfaces are not planar, but randomly distributed throughout the bulk of the material, such as in coatings of spherical Ni particles with Pt nanoparticles. This type of composite approach has multiple advantages over thin-film heterostructures for energy conversion applications, such as the use of scalable manufacturing techniques to prepare bulk quantities of material, leading to potentially more efficient thermoelectric/spin-caloritronic energy conversion overall. This is possible since the combination of SSE and ANE means the entire bulk of the device is actively generating electrical power from all available thermal energy, instead of only a small active layer near the interface(s). 

The search for additional material systems beyond Ni-Pt that may be favorable for SSE+ANE composites points naturally toward NM-FM compounds where one or more constituent elements exhibit strong SOC, such that simple composites can be made readily that contain an FM spin source intimately mixed with SOC-active matrix materials. The enormous spin-Hall angles calculated for elemental Bi and its dilute alloys with Sb [11] suggest their use as the NM. Elemental Bi is chosen here because it is simpler to use than its alloys with Sb, is inexpensive, and is well-known for thermoelectric (TE) applications. A review of known FM compounds immediately suggests that MnBi may satisfy the criteria for the FM. While the magnetic properties of MnBi have drawn interest since at least the 1940s and 1950s [12,13,14], the binary MnBi phase has seen a renewed surge of interest lately due to its potential as a rare earth free permanent magnet. This has resulted in a large body of recent work describing techniques for synthesizing bulk MnBi powders of varying purity with desirable magnetic properties. For example, numerous reports indicate the FM phase has a large remnant magnetization (~70 emu/g) and substantial coercivity (~1T) at room temperature [15,16]. The demonstrated ability to introduce anisotropic microstructural features in MnBi-Bi composites also indicates interesting possibilities for selectively enhancing the materials’ transport properties. 

The binary phase diagram of Mn and Bi [17] shows no solubility of Mn in Bi, though there is a eutectic point at ~4 at.% Mn [18,19]. There are only two equilibrium line-compounds in this system: MnBi, a low-temperature (LT) FM phase; and Mn_1.08_Bi, a high-temperature (HT) paramagnetic phase. Neither melts congruently [20]. The LT phase is stable up to its peritectic decomposition temperature of 355 °C, where it breaks down into liquid Bi with Mn in solution mixed with the HT Mn_1.08_Bi phase. The HT phase is bounded below by a peritectoid decomposition at 340 °C into Mn metal and the LT MnBi phase. Above the peritectic decomposition temperature at 446 °C, the HT phase melts into liquid Bi with Mn in solution and solid Mn. While there are several previous studies of MnBi-Bi composites with compositions at or near the eutectic, the location of the liquidus line indicates that, under equilibrium conditions below ~355 °C, Mn-Bi alloys with Mn content up to ~10 at.% are expected to contain phase-pure MnBi particles precipitated from solution. The relatively gradual slope of the liquidus line further suggests that MnBi precipitates are likely to be present in alloys with higher Mn content if the materials are processed under non-equilibrium conditions (e.g., by quenching and subsequent annealing). However, to our knowledge two reports from Liu and colleagues [21,22] are the only other studies that have explored the properties of alloy compositions far from the eutectic point.

For this study, we used a synthesis strategy of melt quenching followed by annealing to make several samples of Bi containing MnBi precipitates with varying Mn content. Previous studies [23,24] have shown that annealing in a magnetic field tends to produce elongated MnBi needles embedded within the Bi matrix. This strongly textured microstructure results in highly anisotropic magnetic behavior, including remnant magnetization [18,19]. Here, microstructural characterization revealed that samples contained only Bi and MnBi, indicating a complete reaction of metallic Mn. Although the sizes and shapes of MnBi grains varied with composition, they were observed to be universally oriented, with their c-axis along the direction of applied annealing field. There was an optimal composition (10 at.% MnBi) at which the MnBi inclusions primarily formed needle-like grains with very high length-to-width aspect ratios, resulting in a corresponding maximum geometrical demagnetization factor; however, it was the 2 at.% MnBi sample that had the largest SSE-assisted boost to the Nernst coefficient. While the magnetic anisotropy assisted the formation and propagation of magnons in the MnBi, the surface area of the MnBi inclusions was the stronger determining factor of the optimal composition. In conjunction with microstructural characterization, we also measured the anisotropic resistivity, Seebeck, and Nernst effects of a range of MnBi concentrations to search for evidence of a contribution from the SSE to the materials’ transport properties.

## 2. Materials and Methods

### 2.1. Material Synthesis

Composites of MnBi in a Bi matrix were fabricated in a multi-step process. First, 99.999% pure Bi (5N Plus Inc., St. Laurent, QC, Canada) was mixed with 99.95% pure Mn powder (Alfa Aesar, Tewksbury, MA, USA) with a mesh size of −325 in various proportions in a glovebox under an argon gas environment to minimize oxidation of Mn. The mixtures were vacuum-sealed in quartz ampules (Quartz Scientific, Fairport Harbor, OH, USA) and placed in a furnace (ThermoFisher Scientific, Waltham, MA, USA) for approximately 16 h at 700–1000 °C, with the maximum temperature adjusted based on the Mn content to ensure complete melting according to the phase diagram [17]. After 16 h in the furnace, the ampules were water-quenched to convert the melted contents to solid composites while bypassing the formation of solid Mn metal. To promote phase purity and align the MnBi grains, the ampules were then placed in a homemade magnetic annealing apparatus where they were exposed to a 1.4 T magnetic field and heated to 230 °C for approximately 16 h. The ampules were then broken open and the ingots were cut using a low-speed rotary saw (Buehler, Lake Bluff, IL, USA) into multiple pieces for further characterization.

### 2.2. Composition Characterization

**Figure 1:**
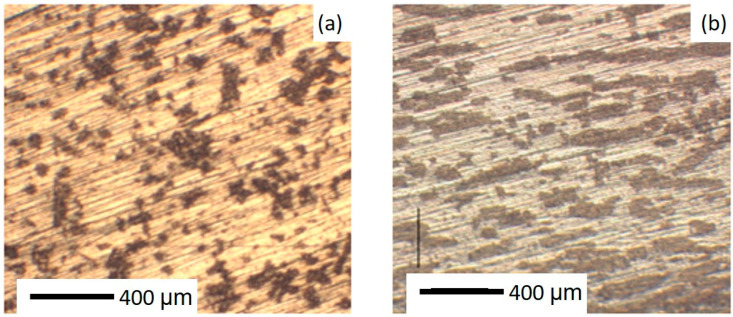
Optical micrographs of a sample of 35 at.% MnBi in a Bi matrix: (**a**) prior to the magnetic anneal; (**b**) after the magnetic anneal. The MnBi are the dark areas, while the lighter areas are the Bi matrix. The annealing process induces a shift from apparently equiaxial to obviously elongated MnBi grains.

To understand the distribution and orientation of the MnBi and Bi grains, microstructural analysis was conducted on several samples of various MnBi compositions: 35, 20, 10, 2, and 1 at.%, using electron and/or optical microscopy. Figure 1 shows optical micrographs illustrating the effect of the magnetic annealing on the shape of the MnBi precipitates in a sample with 35 at.% MnBi after a rough surface polish. The MnBi precipitates take an elongated form after magnetic annealing. We show next that this elongated form can become quite needle-like at an optimum MnBi concentration.

Electron microscopy characterization was performed at the Ohio State University (OSU) Center for Electron Microscopy and Analysis (CEMAS) on mechanically polished samples using an Apreo LoVac Scanning Electron Microscope (SEM) (ThermoFisher Scientific, Waltham, MA, USA). Energy dispersive X-ray spectroscopy (EDS) data were collected with an Octane Elect Plus detector (EDAX LLC, Mahwah, NJ, USA), and electron backscatter diffraction (EBSD) data were collected with aHikari Super camera (EDAX LLC, Mahwah, NJ, USA). EBSD pattern files were saved and re-indexed using the EDAXOIMA software (*Orientation Imaging Microscopy Analysis*, EDAX LLC: Mahwah, NJ, USA, 2016) with Neighbor Pattern Averaging (NPAR).

**Figure 2:**
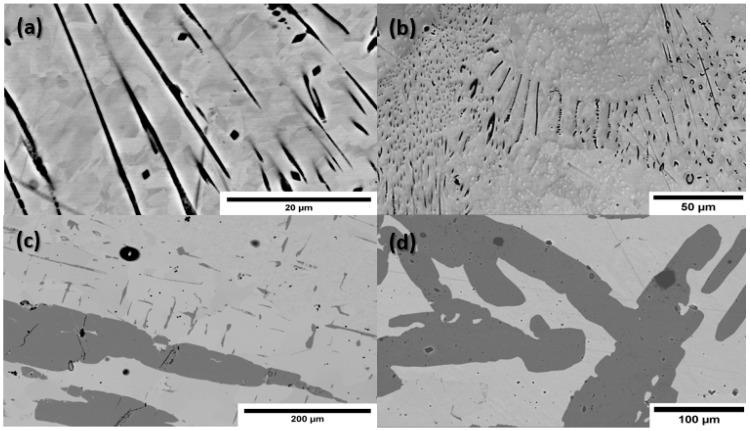
Representative backscatter electron images of four different MnBi-Bi composite samples: (**a**) 1 at.%; (**b**) 2 at.%; (**c**) 20 at.% magnetically annealed; (**d**) 20 at.% unannealed. The darker features correspond with MnBi grains and the brighter areas are the Bi matrix. Needle-like structures can be seen in the low Mn content samples, while elongated polycrystals of MnBi are observed in the 20 at.% samples. The random orientation of the MnBi grains in the unannealed sample is evident in the rounded edges and inconsistent elongation direction of the grains.

**Figure 3:**
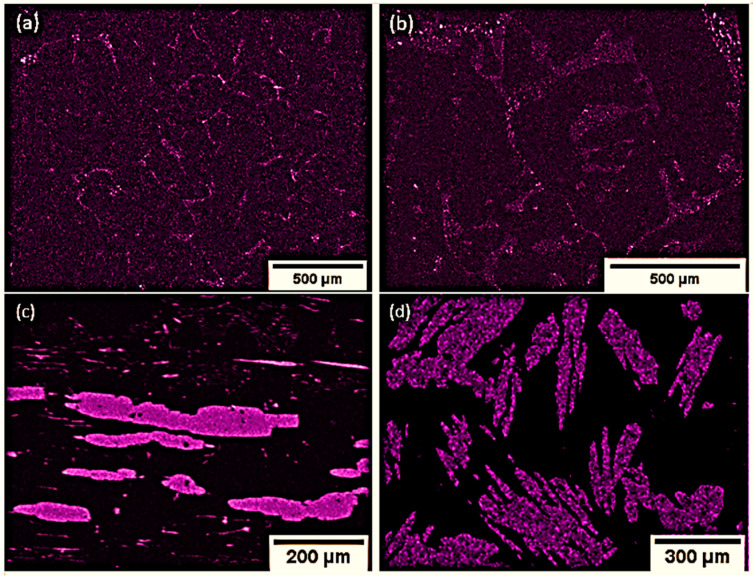
Representative energy dispersive spectroscopy (EDS) maps showing the distribution of Mn within samples of various compositions: (**a**) 1 at.%; (**b**) 2 at.%; (**c**) 20 at.% magnetically annealed; (**d**) 20 at.% unannealed. Image contrast has been enhanced digitally for clarity. Bi maps (not shown) reveal Bi is present everywhere Mn is observed, indicating a complete reaction of all available Mn and confirming that the dark areas in backscatter electron (BSE) images in Figure 2 are MnBi. Samples with low Mn content indicate MnBi is mainly clustered at boundaries between Bi grains, while nearly percolated MnBi networks form in samples with higher Mn content, which are randomly oriented in the unannealed samples.

Representative backscatter electron (BSE) images collected via SEM are shown in Figure 2 for samples of various compositions. EDS maps of the Mn distribution are provided in Figure 3, which confirm that the darker features in the BSE images are MnBi grains. From the BSE and EDS images, we see a general trend emerge in the microstructure; for samples with Mn content below the eutectic composition, the MnBi grains are present in the form of thin needles and small clusters, predominantly at the boundaries between larger Bi grains. For samples with Mn content above the eutectic composition, we observe large, elongated polycrystalline MnBi grains, which form a nearly percolated network at and above 20 at.% MnBi.

To elucidate the effect of annealing in a magnetic field, we also examined the microstructure of a 20 at.% MnBi sample that was not annealed in field. Resulting BSE and EDS data are included in Figure 2 and Figure 3, where elongated polycrystalline grains are apparent, but are generally more rounded in shape (Figure 2), and are elongated in random directions (Figure 3), indicative of no preferred orientation for their growth. This conclusion is further validated via a texture analysis of EBSD data (not included).

**Figure 4:**
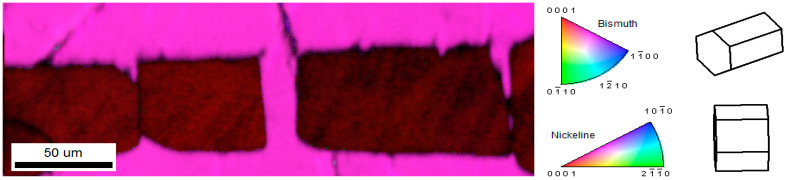
Representative electron backscatter diffraction (EBSD) inverse pole figure (IPF) [010] map (colorized legend to the right of image) overlayed on a greyscale image quality map of the magnetically annealed 20 at.% MnBi sample. The darker red rectangle features are MnBi, which adopts the hexagonal nickeline structure. The consistent red color of the grains indicates they are all almost perfectly oriented with their c-axis along the left–right A2 axis. The c-axis of the surrounding Bi grain in which they are embedded is oriented at approximately 24° relative to the A2 axis, with the surface normal plane being $2\bar{4}29$.

A representative EBSD inverse pole figure map obtained from a 20 at.% MnBi sample annealed in field is included in Figure 4, where we see parts of four distinct MnBi crystallites embedded in a single larger grain. These and similar EBSD data collected on samples of other compositions provide direct evidence for both the polycrystalline nature of the elongated grains, as well as the nearly perfect alignment of their c-axes along the direction of applied field. We note that even the smaller needle-like structures seen projecting from the larger grain in Figure 2c were confirmed to be aligned with their c-axis in the same direction as the applied field, indicating these needle structures are actually elongated along the a-b plane.

### 2.3. Magnetic Characterization

**Figure 5:**
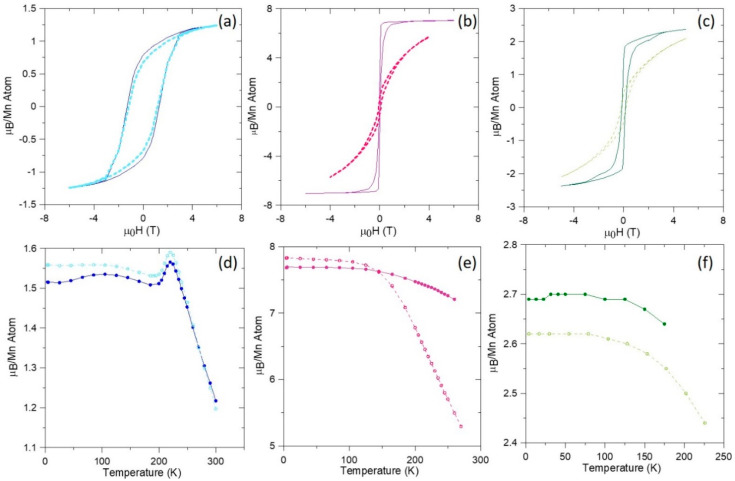
Magnetization versus field at 300 K and moment per Mn ion versus temperature at 5 T, respectively, of samples with the following compositions: (**a**,**d**) 2 at.% MnBi; (**b**,**e**) 10 at.% MnBi; (**c**,**f**) 35 at.% MnBi. Traces were taken with the field applied parallel (full curves, darker colors) and perpendicular (dashed curves, lighter colors) to the direction along which the annealing field was applied.

Magnetization measurements were conducted on the samples using a superconducting quantum interference device (SQUID) in a Quantum Design Magnetic Property Measurement System (Quantum Design, San Diego, CA, USA). Figure 5 shows hysteresis curves of the 2, 10, and 35 at.% MnBi samples at room temperature. The curves are given with the measurement field aligned parallel or perpendicular to the direction along which the field was applied during the magnetic anneal; for simplicity, this direction will be labeled further with the direction of the “needles”, even in samples in which there is little evidence of needle formation. The orientation of the needles appears to have had little effect on the magnetic properties of the 2 at.% sample, suggesting less overall anisotropy of the MnBi grains. This is consistent with the microstructural features seen in Figure 3a,b. Although some MnBi needle-like structures are observed, most of the magnetic inclusions appear in the form of spherical particles clustered at the Bi grain boundaries. The magnetic anisotropy is very pronounced in the 10 at.% MnBi sample at 300 K, which is consistent with microstructural analysis indicating formations of large but isolated needle-shaped inclusions in the sample. The 35 at.% sample has a much lower anisotropy, which is consistent with the more rounded shapes of the MnBi grains observed in Figure 1 and in the 20 at.% sample in Figure 3.

The temperature dependence of the magnetic moment per Mn ion mirrors the general trends seen in the hysteresis curves. The 2 at.% MnBi sample shows a peak at approximately 220–240 K for both needle orientations, again with little evidence of anisotropy. The 10 at.% samples shows substantial anisotropy at higher temperatures, though without the peak near 220 K, and the 35 at.% sample shows an extent of anisotropy intermediate to the other two compositions.

To understand the origin of the peak around 220 K in Figure 5d, we consider that single-crystal MnBi undergoes a spin-reorientation transition between 140 and 90 K, in which the Mn magnetic moments shift from pointing along the c-axis to pointing in the a-b plane [23] upon cooling. Here, we hypothesize that the Bi matrix exerts a pressure on the inclusions that restricts this transition, such that the phase change happens only once the thermal energy is high enough to assist the transition. Further experiments are needed to confirm this explanation.

### 2.4. Transport Measurements

**Figure 6:**
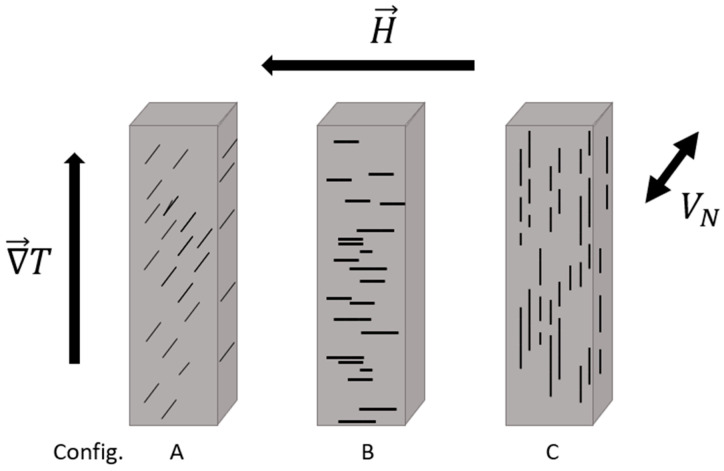
Illustration of the three different cuts made from each sample vis-à-vis the direction of the magnetic field applied during the magnetic anneal, labeled as the MnBi “needle” direction: (**A**) needles are parallel to the Nernst voltage; (**B**) needles are parallel to the applied magnetic field; (**C**) needles are parallel to the temperature gradient. In the longitudinal measurements (resistivity and thermopower), the current, temperature gradient, and induced voltages are all parallel to the long axis of the parallelepipeds. Nernst measurements are made with ∇*T* and *H* applied and the Nernst voltage (*V*_N_) measured as indicated. Given MnBi’s spin-orientation behavior, a Nernst measurement in configuration A corresponds to the traditional spin-Seebeck effect (SSE) geometry below 90 K and configuration B above 140 K. Configuration C was used as a control.

The materials’ longitudinal and transverse transport properties were measured in a conventional five-probe geometry, which required first cutting the samples using a low-speed diamond-tipped rotary saw (Buehler, Lake Bluff, IL, USA) into rectangular prisms with approximate dimensions of 1.5 × 1.5 × 5 mm^3^. Conductive silver epoxy (EpoTek, Bellerica, MA, USA) was used to affix each sample with copper-constantan thermocouples and copper electrical leads (Omega Engineering, Norwalk, CT, USA), with an alumina (MTI Corporation, Richmond, CA, USA) heat sink on one end and a resistive heater (Omega Engineering, Norwalk, CT, USA) on the other. Measurements were taken using a Janis cryostat (Lake Shore Cryotronics, Westerville, OH, USA) between temperatures of 80 and 400 K. The samples were positioned carefully in the cryostat to ensure the desired orientation of the magnetic field and temperature gradient during each set of measurements. These orientations are depicted in Figure 6 and denoted as: (A) needles perpendicular to both field and temperature gradient; (B) needles aligned with the applied field while the temperature gradient was mutually perpendicular; and (C) needles aligned with the temperature gradient while the applied field was mutually perpendicular. At each temperature point, a steady temperature gradient was established for a minimum of 20 min before the transverse voltage was recorded at various magnetic field values between −1.4 and 1.4 T.

## 3. Results

**Figure 7:**
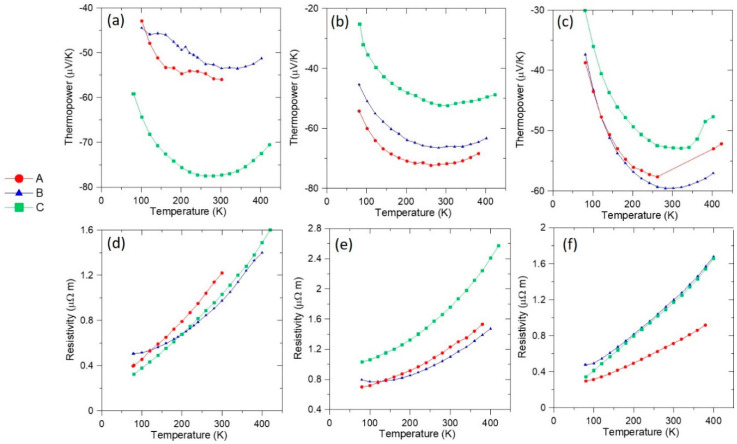
The top row shows temperature dependence of the thermopower of samples in each configuration (see Figure 6) with the following compositions: (**a**) 2 at.% MnBi; (**b**) 10 at.% MnBi; (**c**) 35 at.% MnBi. The bottom row shows resistivity versus temperature of samples in each configuration with the following compositions: (**d**) 2 at.% MnBi; (**e**) 10 at.% MnBi; (**f**) 35 at.% MnBi.

Figure 7a–c displays the zero-field longitudinal thermopower and Figure 7d–f displays the resistivity as a function of temperature for the three configurations shown in Figure 6. We note that at zero field, configurations A and B should be equivalent for any longitudinal property measurement. Considering first the 2 at.% sample, we see a surprisingly large variation between the thermopower in different configurations; the data for configuration C are close to the average between the in-plane and c-axis thermopower of elemental Bi [25], as expected for a polycrystalline Bi matrix. Application of the effective medium theory would predict that thermopower of a dilute random composite with spherical inclusions should not differ much from that of the matrix [26]. Here, we see small differences between configurations A and B. This could be explained by considering the micrographs in Figure 3a,b, which reveal the metallic MnBi inclusions are not distributed randomly, but instead form preferentially orientated chains along the grain boundaries that effectively short out the thermopower of the matrix. In the 10 and 35 at.% samples, the thermopower in configuration C is the lowest, consistent with the thermopower of the matrix now being short-circuited by the large and (nearly) percolated MnBi needles.

**Figure 8:**
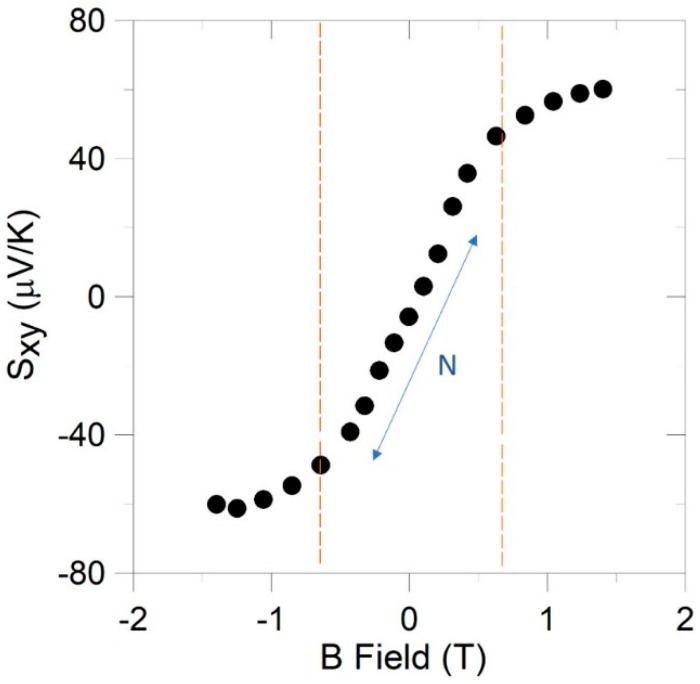
An example of the magnetic field dependence of the Nernst thermopower in the 50 at.% sample in B configuration at 80 K. The curve shows two different regimes, a low field (|*B*| < 0.6 T) and a high field (|*B*| > 1 T). The slope is taken at the low field then normalized by the temperature gradient and sample dimensions to calculate the intrinsic Nernst coefficient.

The Nernst thermopower is defined as $S_{xy}=E_{y}/\nabla_{x}T$, with *x* the direction of the applied gradient in Figure 6 and *y* the direction of *V*_N_, from which the Nernst coefficient is then defined as the slope of $S_{xy}$ near the zero field, $N=dS_{xy}/dB$. Figure 8 shows an example of Nernst thermopower data for the 50 at.% sample in configuration B at 80 K as a function of magnetic field for |*B*| < 0.6 T. Given the differences in needle orientation and voltage measurement direction between different configurations, comparison of Nernst coefficient data collected in these geometries allows for isolation of any SSE contribution to *N*.

**Figure 9:**
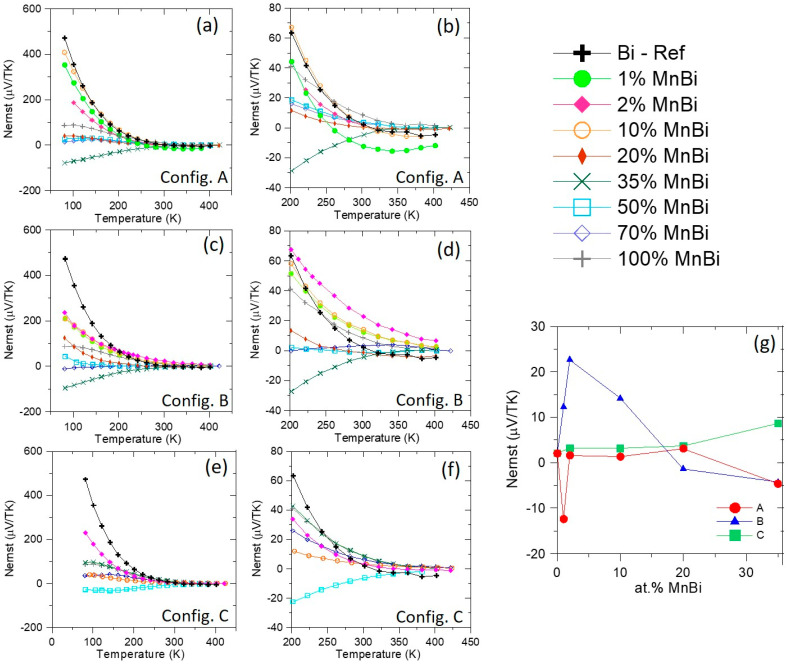
Low-field (*|B|*< 0.6 T) Nernst coefficients of the MnBi compositions listed in the legend in the upper right corner in the following configurations: (**a**) configuration A at all temperatures; (**b**) configuration A at higher temperatures; (**c**) configuration B at all temperatures; (**d**) configuration B at higher temperatures; (**e**) configuration C at all temperatures; (**f**) configuration C at higher temperatures. (**g**) shows the Nernst coefficient of each configuration as a function of MnBi composition up to 35 at.%, approximately the percolation limit. We note an enhancement of the Nernst coefficient at T > 220 K for most composite samples in all three configurations when compared to the value of pure Bi, but that enhancement is much greater in configuration B, where an SSE contribution is expected, rather than in A or C.

Figure 9 shows the Nernst coefficient for the A (Figure 9a), B (Figure 9c), and C (Figure 9e) configurations with an additional plot emphasizing the behavior at higher temperature data points (Figure 9b,d,f, respectively). Here, we expected to see a boost in the ANE above 140 K in the B configuration, relative to the pure Bi sample. As shown in Figure 9a,c, the Nernst coefficient of the Bi sample is larger than that of the MnBi-containing samples at almost all temperatures below about 220 K. Above 220 K, we see an enhancement in the Nernst coefficient for the 1, 2, and 10 at.% MnBi samples in configuration B, where an SSE contribution is expected (see Figure 6), but no enhancements in configurations A or C. An additional plot is provided in Figure 9g showing the Nernst coefficient of the composite samples at 300 K up to 35 at.% MnBi, which appears to be the percolation limit for our samples. The magnitudes of the Nernst coefficient in the 1, 2, and 10 at.% MnBi composites in the B configuration are about 6, 11, and 7 times larger, respectively, than the pure Bi reference sample at room temperature. Smaller enhancements are observed in configurations A and C. This leads us to conclude that an SSE contribution, expected in configuration B only, is indeed present. Surprisingly, even the 35 at.% MnBi sample in the C configuration is almost four times higher than the Bi reference sample.

The values of *N* observed below 220 K in MnBi-containing samples fall in between the values measured on elemental Bi and pure MnBi, suggesting some type of effective medium behavior for these composites. We are not aware of any effective medium theory for the Nernst coefficient, so this hypothesis cannot currently be explored further. More interesting is the enhancement of *N* observed in the dilute MnBi-containing samples over the elemental Bi sample above 220 K (Figure 9b,d), especially the 2 at.% MnBi sample in the B configuration. The surface area shared between the MnBi and Bi is large, as observed in Figure 3b, and it follows that this would lead to an enhancement in the transverse voltage as SSE is an interfacial effect. As the MnBi content increases, the magnetic anisotropy also increases, but larger MnBi grains means the shared surface area decreases. This behavior manifests as a large increase in the ANE in the 10 at.% MnBi sample, indicating a boost from the magnetic anisotropy from formation of sharp MnBi needle structures. This is unlikely to come from any effective medium contribution, since *N* is larger in the composite than in any constituent. Instead, the most likely explanation is that this enhancement arises from an SSE contribution to the Nernst effect in this oriented composite material system.

## 4. Conclusions

SSE and Nernst can be combined in bulk materials to amplify thermal effects. Here, we successfully synthesized a series of composite materials with aligned MnBi needles in a Bi matrix. This was done by heating Mn and Bi powder in a furnace and water-quenching, followed by annealing the samples in a magnetic field to promote alignment of MnBi grains. We observed evidence of a possible shift in the spin-reorientation temperature of MnBi at 220 K when embedded in a composite. More importantly, we observed that the length-to-width aspect ratio of the MnBi particles was optimized for the sample containing 10 at.% MnBi, in which the MnBi formed needle-like shapes within the matrix that corresponded with maximized magnetic anisotropy at higher temperatures. Above 220 K, Nernst measurements suggest that a shared surface area and magnetic anisotropy are likely the two most important parameters governing the extent of any SSE contribution to the Nernst voltage. Further study of SSE contributions to transverse thermopower in magnetically aligned composites with optimized microstructures may lead to additional enhancements.
